# Intracellular calcium signaling and phospho-antigen measurements reveal functional proximal TCR activation in lymphocytes from septic shock patients

**DOI:** 10.1186/s40635-019-0287-5

**Published:** 2019-12-23

**Authors:** Charles de Roquetaillade, Khalil Kandara, Morgane Gossez, Estelle Peronnet, Céline Monard, Martin Cour, Thomas Rimmelé, Laurent Argaud, Guillaume Monneret, Fabienne Venet

**Affiliations:** 1 EA 7426, Pathophysiology of Injury-Induced Immunosuppression (Université Claude Bernard Lyon 1 - Hospices Civils de Lyon - bioMérieux), Edouard Herriot Hospital, 69437 Lyon, France; 2 Current address: INSERM U942 MArkers in Stressed COndiTions (MASCOT), Hôpital Lariboisière, Lyon Cedex, 03 Paris, France; 3 Immunology Laboratory, Hôpital E. Herriot - Hospices Civils de Lyon, 69437 Lyon, France; 4 Joint Research Unit HCL-bioMérieux, Hôpital Edouard Herriot, 5 place d’Arsonval, 69003 Lyon, France; 5 Anesthesia and Critical Care Medicine Department, Hospices Civils de Lyon, Edouard Herriot Hospital, 69437 Lyon, France; 6 Intensive Care Medicine Department, Hospices Civils de Lyon, Edouard Herriot Hospital, 69437 Lyon, France

To the editor,

Sepsis deeply perturbs immune homeostasis by inducing a complex immune response that varies over time and associates a tremendous systemic inflammatory response to anti-inflammatory mechanisms. As a delayed consequence, some septic patients enter a state of profound immunosuppression [1]. As the latter may persist for weeks, leaving the patient at increased risk of secondary infections, immunostimulation recently appeared as a reasonable therapeutic option in patients with signs of persistent and severe immunosuppression [2].

Septic patients develop marked T lymphocyte dysfunctions such as profound lymphopenia, increased expression of inhibitory co-receptor molecules, decreased repertoire diversity, and reduced functionality (proliferation and cytokine production). These alterations have been repeatedly associated with deleterious outcomes [1, 2]. However, mechanisms leading to these alterations are only partially understood. For instance, while the role of deactivated mTORC1 is established [3], the intrinsic capacity of T cell receptor (TCR) to be activated and to transduce intracellular signalling remains unexplored. Among determinants of T cell response, immediate calcium signaling following TCR ligation is of paramount importance and serves crucial effector functions. Thus, we developed a flow cytometry protocol to follow calcium flux after TCR stimulation in patients’ CD4+ T lymphocytes. In addition, phosphorylation of molecules from the proximal and downstream TCR signaling cascade was analyzed (Additional file 1). We included patients with septic shock (according to SEPSIS-3 definition) presenting with features of immunosuppression, i.e., decreased monocyte HLA-DR and lymphocyte count (clinical and immunological characteristics in Additional file 1: Table S1).

We show that immediate signaling downstream TCR stimulation was not altered in circulating CD4 lymphocytes from septic shock patients. Indeed, cells exhibited no deregulation of intracytoplasmic calcium influx after TCR ligation compared with healthy controls (OKT3 response, Fig. 1). In agreement, we observed a significant CD3ζ phosphorylation (one of the first molecules to be phosphorylated after TCR engagement) after T cell stimulation in both cells from septic patients and controls (Fig. 2). This showed that immediate response after TCR activation was unaffected after sepsis.

**Fig. 1:**
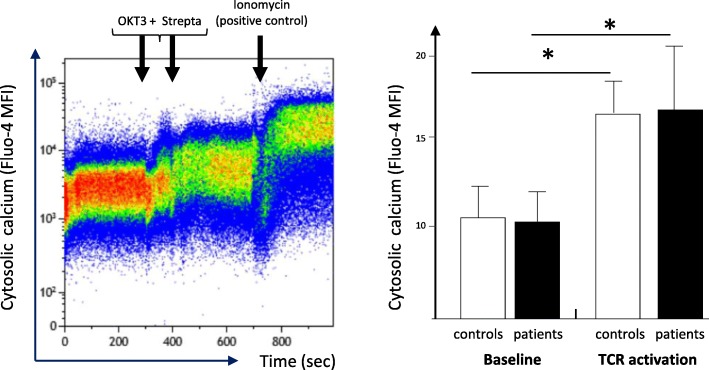
Intracellular calcium signaling in CD4+ T cells from septic patients and controls upon TCR ligation. Peripheral blood mononuclear cells (PBMC) were isolated from patients and controls and loaded with Fluo-4AM for 30 min at 37 °C. Cells were analyzed by flow cytometry for 5 min at baseline then stimulated by biotinylated anti-CD3 (OKT3) antibody during 90 s before addition of streptavidin to induced TCR stimulation and analyzed for another 5 min. As a positive control, ionomycin was added at the end of experiment. Left, representative example of overtime intracellular calcium staining in one healthy volunteer. Right, intracellular calcium staining following TCR ligation in septic patients (*n* = 7) and healthy volunteers (controls, age-matched, *n* = 7) before (baseline), after TCR stimulation (biotinylated anti-CD3 antibodies + streptavidin) and after ionomycin addition. MFI of Fluo-4AM was measured in CD4+ T lymphocytes on 3 periods of 100 s at baseline and after complete TCR stimulation. For each stimulation condition, the maximum MFI was considered. Data are represented as means +/− SD. *p < 0.05 compared to baseline, Wilcoxon paired test

**Fig. 2:**
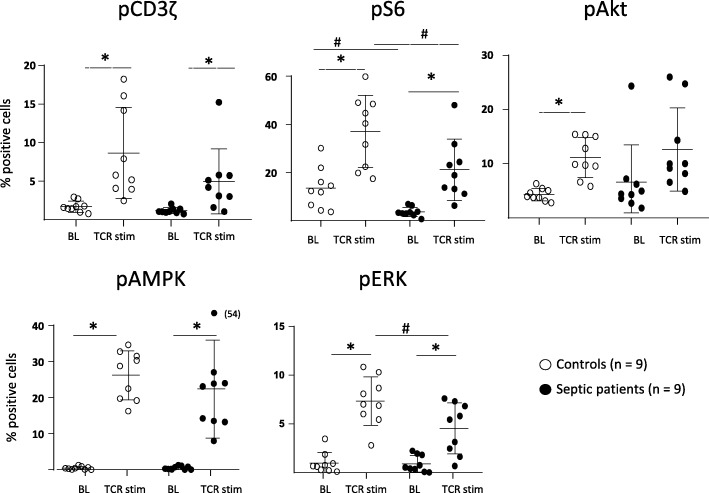
PI3/Akt/mTOR pathway activation in CD4+ T cells from septic patients and controls upon TCR ligation. PBMCs were isolated from septic patients and controls, stimulated for 7 min with anti-CD2-CD3-CD28 Ab-coated beads (bead/cell ratio = 3/1) and stained with anti-CD4 and anti-CD3z, anti-pS6, anti-pAkt, anti-pERk, and anti-pAMPK antibodies protein phosphorylation was assessed by flow cytometry. Data represent the percentage of positive cells based on FMO staining and as individual values and means +/− SD in 9 septic patients and 9 healthy volunteers (controls) before (baseline, BL) and after TCR stimulation (TCR stim). *p < 0.05 (Wilcoxon paired test, baseline vs TCR stim), #*p* < 0.05 (Mann-Whitney, healthy volunteers vs patients after stimulation)

In contrast, activation of more distal molecules in the TCR signaling cascade was impacted. For example, stimulation-induced rise of pAkt and pERK was affected leading to a limited mTORC1 activation capacity as measured by S6 phosphorylation after stimulation. In contrast, activation of AMPK, an inhibitor of mTORC1 was mostly unaltered in patients compared to controls (Fig. 2).

In conclusion, the present results obtained in septic shock patients show that proximal TCR signaling remains functional in circulating CD4+ T cells from septic shock patients while downstream activation of mTORC1 pathway is markedly diminished. PI3K-Akt pathway integrates signals from both co-activating/inhibitory receptors and an increased expression of such co-inhibitory receptors has been described on circulating T cells from septic patients [4]. In that respect, this suggests that inhibitory receptors known to block downstream signaling are likely of utmost importance in sepsis-induced T lymphocyte dysfunctions. As TCR from septic lymphocytes remains actionable [5], the present results reinforce the rational for blocking co-inhibitors (e.g., with anti-PD-1) or stimulating mTORC1 (for example with rhIL-7) as reasonable immunoadjuvant approaches to tackle sepsis-induced immunosuppression [6–8].

## Supplementary information

**Additional file 1: Table S1.** Clinical and biological data from septic shock patients.

## Data Availability

The datasets used and/or analyzed during the current study are available from the corresponding author on reasonable request.

## Abbreviations

anti-PD-1: Anti-Program-Death-1
HLA-DR: Human leukocyte antigen–DR isotype
mTORC1: Mammalian target of rapamycin complex 1
OKT3: Monoclonal antibody anti-CD3 receptor (muromomab)
pAkt: Phosphor-Akt (aka phospho-protein kinase B)
pAMPK: Phosphor-mitogen-activated protein kinases
pERK: Phosphor-extracellular signal-regulated kinases
rhIL-7: Recombinant human interleukin-7
TCR: T cell receptor
