# Supplementary material for: Intracellular calcium signaling and phospho-antigen measurements reveal functional proximal TCR activation in lymphocytes from septic shock patients
Source: Intensive Care Med Exp. 2019 Dec 23;7:74. doi: 10.1186/s40635-019-0287-5 (PMC6928172; doi:10.1186/s40635-019-0287-5)

Online supplemental material

**Intracellular calcium signalling and phospho-antigen measurements reveal functional proximal TCR activation in T lymphocytes from septic shock patients**

Charles de Roquetaillade *^1,2^, Khalil Kandara *^1,3^, Morgane Gossez ^1,3^, Estelle Peronnet^1,4^, Céline Monard ^5^ , Martin Cour ^6^, Thomas Rimmelé ^1 ,5^, Laurent Argaud ^6^,

Guillaume Monneret ^1,3^ and Fabienne Venet ^1,3^

1- EA 7426 « Pathophysiology of Injury-Induced Immunosuppression » (Université Claude Bernard Lyon 1 - Hospices Civils de Lyon - bioMérieux), Edouard Herriot Hospital, 69437 Lyon, France

2- Current address: INSERM U942 “MArkers in Stressed COndiTions (MASCOT)“, Hôpital Lariboisière, Paris, France

3- Hospices Civils de Lyon, Edouard Herriot Hospital, Immunology Laboratory, 69437 Lyon, France

4- Joint Research Unit HCL-bioMérieux, Hôpital Edouard Herriot, 5 place d’Arsonval, 69003, Lyon, France

5- Hospices Civils de Lyon, Edouard Herriot Hospital, Anesthesia and Critical Care Medicine Department, 69437 Lyon, France

6- Hospices Civils de Lyon, Edouard Herriot Hospital, intensive Care Medicine Department, 69437 Lyon, France

* these two authors contributed equally to the work

**Table S1.** Clinical and biological data from septic shock patients

For clinical parameters, categorial data are presented as numbers of cases and percentages of the total population in brackets. Continuous data and biological parameters are presented as medians and interquartile ranges [Q1-Q3]. SAPS II (Simplified Acute Physiology Score II) was calculated on admission. SOFA (Sequential Organ Failure Assessment) score was measured after 24h of ICU stay. mHLA-DR (AB/C): number of anti-HLA-DR antibodies bound per monocyte.

|  | Patients (n=16) |
| --- | --- |
| Age (years) | 69.50 [67- 78] |
| Sexe, (female) | 6 (37.5) |
| Charlson comorbidity score | 2.00 [1.25- 3.00] |
| SAPS II score | 70.00 [56.75- 81.50] |
| SOFA score | 10.00 [8.00-10.50] |
| Lactate on admission, mmol/L | 2.80 [2.55- 3.80] |
| **Type of admission** |  |
| medical | 5 (31.25) |
| surgery | 11 (68.75) |
| **Microbiological documentation** |  |
| Gram-negative | 6 (37.5) |
| Gram-positive | 4 (25) |
| Unknown | 6 (37.5) |
| **Outcomes** |  |
| ICU-acquired infection | 2 (12.5) |
| ICU mortality | 4 (25) |
| Day 28 mortality | 5 (31.25) |
| **Biological characteristics at day 3** |  |
| Lymphocyte count, cells/mm^3^ | 990 [820- 1500] |
| mHLA-DR (AB/C) | 4116 [2258- 7503] |
| CD4+ T lymphocytes, cells/mm^3^ | 335 [273- 447] |

**MATERIAL AND METHODS**

**Patients**

This study was conducted in the intensive care units (ICU) of Hôpital Edouard Herriot (Lyon, F). Septic shock patients were identified based on sepsis-3 definition. Blood samples were collected at day 3–4 (D3) after the onset of shock (EDTA-tubes). This project was approved by the Institutional Review Board for ethics (Comité de Protection des Personnes Sud-Est II, number 11236) This study is registered with the French Ministry of Research and Teaching (#DC-2008-509) and with the Commission Nationale de l’Informatique et des Libertés (CNIL) and on clinicaltrials.gov (NCT02803346). Oral information and non-opposition to inclusion in the study were mandatory and recorded in patients’ clinical files. Exclusion criteria disqualified patients younger than 18 years of age, subjects with aplasia or immunosuppressive disease (e.g., HIV infection). Patients showing no feature of sepsis-induced immune suppression (assessed by monocytic HLA-DR (mHLA-DR) inferior to 10 000 AB/C) were also excluded. Blood samples from age-matched healthy volunteers were obtained from Etablissement Français du Sang (EFS, blood bank of Lyon). According to EFS standardized procedures for blood donation and to provisions of the articles R.1243–49 and following ones of the French public health code, a written non-opposition to the use of donated blood for research purposes was obtained from HV. The blood donors’ personal data were anonymized before transfer to our research laboratory.

**Calcium flux analyses**

Mobilization of intracellular calcium was measured by using a flow cytometric assay. Briefly, Peripheral Blood Monuclear cells (PBMCs) were purified using Ficoll gradient-centrifugation (Biochrom GmbH, Berlin, Germany), contaminating neutrophils were depleted using the Granulocyte Depletion Cocktail through the centrifugation (RosetteSep™, StemCell Technologies, Grenoble, France) and the RBCs lysed by the Versalyse solution (Beckman Coulter, Hialey, FL, USA). PBMC isolated from septic shock patients and controls were stained with anti-CD4-PB (Pacific Blue, Beckman Coulter). Two million cells were resuspended in 300 µL of medium (RPMI with 25 mM HEPES + 0.2 % BSA) and incubated with Fluo-4, acetoxymethyl ester (2µM) calcium-sensing dye (Invitrogen, Carlsbad, CA, USA) for 30min at 37°c. Cells were washed with RPMI and resuspended in 300µL of medium (RPMI with 25 mM HEPES + 0.2 % BSA). For analysis, cells were acquired by using the time parameter on the BD FACSAria II (Becton Dickinson, San Jose, CA,) and analysed for FL3 fluorescence. The cell flow rate was 1000 cells per second. Baseline calcium flux analysis was performed for 5 minutes. Biotin anti-CD3 (OKT3, 10µg/mL, Invitrogen) was added thereafter and cells analysed for another 90 seconds. Afterwards Streptavidin (10µg/mL, Invitrogen) was added and cells analysed for 5 minutes. Ionomycin (10µM, Sigma-Aldrich, Saint-Louis, MO, USA) was added for 5 minutes at the end of acquisition. MFI of Fluo4 was measured for 3 consecutive periods of 100 seconds after each stimulus. For each period, the maximal MFI among the 3 values was considered.

**Phosphoprotein expression by flow cytometry**

Isolated PBMCs were washed in sterile Phosphate Buffered Saline (PBS, Eurobio, Les Ulis, France) and resuspended in supplemented RPMI culture media without serum AB. The number of cells per well was adjusted to 1.10^6^ cells per milliliter. Cells were allowed to rest for 1 hour and were then stimulated with anti-CD2-CD3-CD28 Ab-coated beads (bead/cell ratio = 3/1) for 7 minutes. Activation was stopped by the addition of cold PBS, and intracellular phosphoprotein expressions were evaluated via intracellular flow cytometry staining using anti-pAkt-Alexa Fluor 647 (clone Ser473), anti-pS6-PB (clone Ser235/236), biotinylated anti-phospho-AMPK (clone Thr172), Streptavidin-PE (all reagents from Becton Dickinson), anti-CD4-PE-Cy7. Because of redundancy in fluorochromes, a second staining was performed on another tube within the same conditions using anti-pERK-AF647 (Alexa Fluor 647, clone Thr202/Tyr204), anti-CD247-AF488 (CD3 zeta, cloneTyr412) and anti-CD4-PB. Permeabilization and fixation were achieved using Cytofix/Cytoperm and Phosflow Perm buffers (Becton Dickinson). Cells were analyzed immediately on Navios (Beckman), gating strategy is described in figure S1.

**Statistical analyses**

Unless otherwise stated, results are expressed as Means +/- Standard deviation (SD). Comparisons between groups (patients vs. healthy donors) were performed with a non-parametric Mann–Whitney U test. Comparisons between different stimulation conditions within the same group were performed with a non-parametric Wilcoxon paired test. Statistical analyses were performed by using GraphPad Prism software (Version 8.0.1). A p value inferior to 0.05 was considered statistically significant.

**Figure S1. Illustrative example of gating strategy** – CD4 lymphocytes are identified on a dot plot histogram (CD4 – SSC, left histogram) after excluding doublets. Afterwards, each phosphoprotein is expressed on monoparametric histogram in which positivity threshold is set up by FMO (blue peak, fluorescence minus one). In unstimulated (green peak) and stimulated (red peak) conditions, results are then expressed as % of positive cells (i.e., cells above the threshold for positivity).


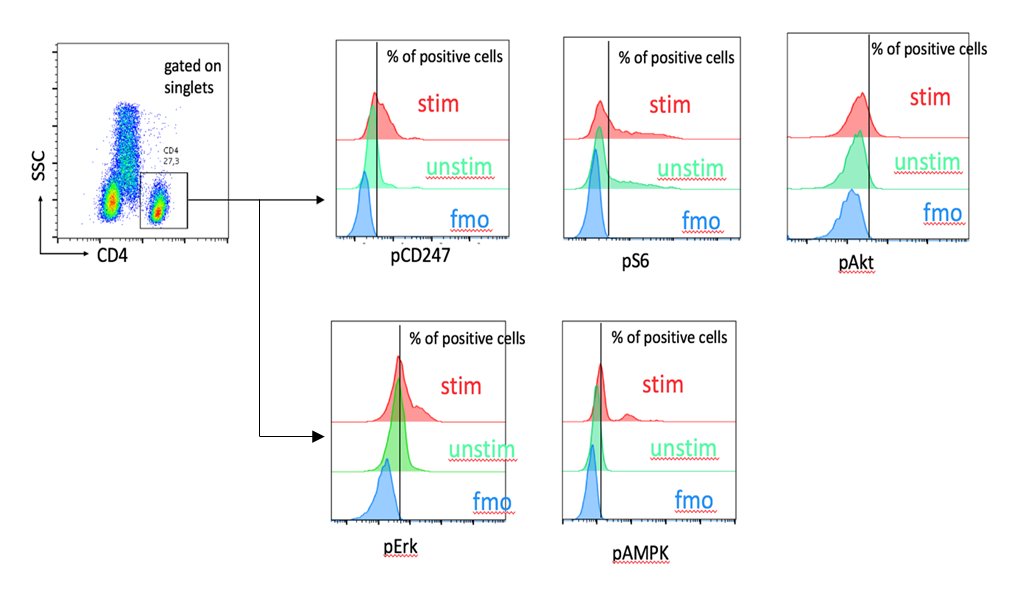

Supplement: Supplementary file 1 — Additional file 1: Table S1. Clinical and biological data from septic shock patients. [file 40635_2019_287_MOESM1_ESM.docx]
